# Evaluation of Salt-Induced Damage to Aged Wood of Historical Wooden Buildings

**DOI:** 10.1155/2020/8873713

**Published:** 2020-08-01

**Authors:** Xiaochen Mi, Tieying Li, Jinping Wang, Yongfeng Hu

**Affiliations:** ^1^Collage of Civil Engineering, Taiyuan University of Technology, Taiyuan 030024, Shanxi, China; ^2^Collage of Architecture, Taiyuan University of Technology, Taiyuan 030024, Shanxi, China; ^3^Canadian Light Source, 44 Innovation Boulevard, Saskatoon, SK S7N 2V3, Canada

## Abstract

Salt is a common cause of damage to building materials used in cultural and historical buildings. The damage to aged wood in historical wooden buildings has not been extensively studied, resulting in the need for a more detailed analysis. In this work, Yingxian Wooden Pagoda, a typical historical wooden structure, was taken as the research object. Multichemical analyses were conducted to evaluate and understand the salt-induced damage to the aged wood using a scanning electron microscope equipped with an energy-dispersive X-ray spectrometer, sulphur K-edge X-ray absorption near-edge structure spectroscopy, X-ray fluorescence spectroscopy, X-ray powder diffraction, and attenuated total reflectance fourier transformed infrared spectroscopy. The results showed the presence of invasive salt crystallisations and ions in the aged samples. The source of these invasive elements was deduced by identifying the type, amount, and valency of the elements; they were found to be derived from environmental factors such as acid rain and atmospheric pollutant. The unique damage mechanism and route induced by salt in historical buildings made of wood were summarised; the damage was attributed to the accumulation of sulphate salt causing hydrolysis of the carbohydrates and salt crystallisation resulting in mechanical damage. This interdisciplinary study is significant for decision making in studies related to the preservation and evaluation of historical wooden buildings.

## 1. Introduction

For centuries, the building materials of cultural buildings have undergone multifaceted damage including mechanical, environmental, and biological degradations. Salt crystallisation is one of the sources of damage [1]. Although these types of damages are universal, the changes do not become obvious in a short time and are easily overlooked.

Salt deterioration of ancient cultural buildings is an important issue worldwide. This is because of the intrusive characteristics of salt and its influence on structural components, threatening the sustainable preservation of our valuable cultural heritage buildings. Salt easily gets deposited in the pores of building materials exposed to the natural environment [2]. The numerous salt crystals formed in the pores through phase change can lead to an increase in the elastic limit of the material [3]. Consequently, the materials undergo severe damage such as discoloration and deposition, flaking, loss of material, and even material disaggregation [4]. These damages are mostly induced at the structural cross sections and gradually migrate inward from the surface, leading to a decrease in the load and deformation capacity of the overall structure and affecting the stability.

Several studies have been conducted on the salt-induced deterioration of rocks, limestone, concrete, and other porous building materials [5–7]. The results show that salt-induced deterioration can lead to significant changes in the physical and mechanical properties of such materials, because of the increase in their porosity and water absorption capacity and decrease in their uniaxial compressive strength, tensile strength, and other mechanical index values [8]. The salt-induced damage to porous materials has been extensively studied, including its damage mechanism, main influencing factors, and development of methods to prevent damage [9–11]. However, such studies on wooden historical buildings are limited, with no clear answer on the effect of salt. Wood has been extensively used as a building material in cultural buildings. Many world-renowned historical buildings are made of wood, such as the Imperial Palace (China), Todaiji Buddha Temple (Japan), and Sungnyemun (South Korea). Wooden historical buildings are widely distributed in the world, and they should be effectively evaluated and protected. Although wood is a porous material, its properties are unlike those of other porous materials such as stone or concrete. As a biomaterial, it has a complex anatomical structure and is composed of wood cell units including three compounds, namely, lignin, hemicelluloses, and cellulose. The type and degree of damage caused by salt depend on several factors such as the properties of the material; type, amount, and source of salt; and environmental conditions [12]. The effects of these factors on wooden historical buildings are unclear.

**Figure 1:**
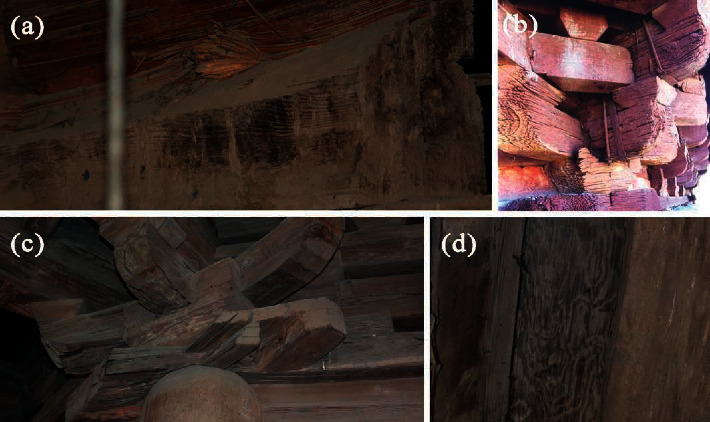
Damages on the surface of wooden components in Yingxian Wooden Pagoda. There are decreased effective bearing areas of (a) beams, (b) Dou-Gong brackets, and (c) smaller Dou brackets, and (d) the surfaces of most columns have lower hardness and density, which are attributed to the decay on the surface of the wood.

The objective of this study was to evaluate the salt-induced damage to Yingxian Wooden Pagoda (Shanxi, China), one of the representative wooden historical buildings, and attempt to understand the nature, cause, and damage mechanism for its preventive conservation. Yingxian Wooden Pagoda was built in 1056 AD and is 67.31 m high, making it the tallest ancient all-wooden structure in the world (details are shown in the supplementary material: Figure S1). Through a thousand years, the surface of the wooden components has been decayed. Components, particularly with a small cross section, such as beams (Figure 1(a)) and Gong brackets (Figure 1(b)), have a larger proportion of decreased effective bearing area due to surface wood damage, leading to their deformation or fracture. Similar damages are also observed in the smaller Dou brackets (Figure 1(c)), causing residual compression deformation. The surfaces of most columns (Figure 1(d)) have lower hardness and density. When pressed by hand, the structure feels soft, and an indentation can be easily made. A fine needle can penetrate to depths in the range of 0.4–1.5 cm from the surface.

In this work, microsamples were taken from the surface of the second to eighth floors of the pagoda by multiple chemical characterisations. Scanning electron microscopy (SEM) was applied to observe the microscopic morphology, and X-ray powder diffraction (XRD) was used to identify crystalline compounds. These were employed to evaluate the salt crystallisation in the aged wood and thus determine the type, amount, and source of the salt. X-ray fluorescence (XRF) was applied to qualitatively and semiquantitatively detect inorganic elements. Subsequently, sulphur K-edge X-ray absorption near-edge structure (XANES) spectroscopy was employed to identify the chemical valency of sulphur. To understand the effects of the degraded conditions of the aged wood, the pH value was measured using a pH meter. Functional group analyses of cellulose and hemicelluloses were carried out using attenuated total reflectance fourier transformed infrared (ATR FT-IR) spectroscopy, and the relative degree of crystallinity (CrI) of cellulose was calculated. The proposed multichemical characterisation has the advantages of low dosage, less destructiveness, reusability of samples, and high accuracy, thus enabling the evaluation of other similar historical wooden buildings. The study results shed new light on the salt-induced damage processes to aged wood, revealing its damage mechanisms and routes. This study is expected to serve as a theoretical basis for the repair and conservation of ancient buildings.

## 2. Materials and Methods

### 2.1. Samples

In this study, a total of eight samples from Yingxian Pagoda were characterised, which had been previously identified as larch (*Larix principis-rupprechtii* mayr) [13]. Samples were taken from the surface of upright pillars from different positions in the second to the ninth layers, in which the pillars were uncoated (details are shown in the supplementary material: Figure S2). Only a few fragments of the aged wood were sampled (using a blade), where some aged wood was already cracked or located in a hidden zone, because of the important cultural, historical, and artistic values of the pagoda. A piece of contemporary, sound wood of the same species was also tested as a reference sample. Considering the limited number of samples, both the aged and sound wood samples were shorn by surgical scissors into sawdust and dried at room temperature. Some parts of the aged and sound samples were cut into a thin section using a razor blade for SEM and ATR FT-IR characterisation.

### 2.2. Methods

SEM was used to investigate the micromorphology of the samples, even though some parts were highly degraded or collapsed. The samples were cut by hand using a razor blade along the longitudinal anatomical direction. They were mounted on a sample platform covered by conducting tape, treated using a critical point dryer (JEOL, JFD-320), and finally gold-coated (JEOL, JFD-1600). SEM characterisation was performed through JSM-6490LV (JEOL, Japan) equipped with an energy-dispersive X-ray spectrometer (EDS, Oxford X-act, UK) to identify the elemental composition of the samples. The SEM analysis was performed at an accelerating voltage of 20 kV and with magnifications ranging from ×150 to ×2000. Thus, the representative electron micrographs of the samples and X-ray spectra of the elements were acquired for an in-depth study of the decay level of the wood cell walls.

The XRD characterisation was performed on a Bruker-AXS type D8-ADVANCE diffraction machine (Bruker, Germany) equipped with Cu-K radiation (*λ* = 0.154056 nm) at 40 kV and 40 mA. All the powder samples were pressed into a cylindrical wood wafer with a 25 mm diameter and 4.3 mm thickness. The data were recorded in the 2*θ* range of 5–45° and with a step size of 0.04°, using a nickel filter with a wavelength of 1.542 Å. The scans were taken at 5° per min (0.5 s per step). The relative degree of crystallinity (CrI) was calculated using the Segal method [14]:

(1) $$\begin{array}{c} \mathrm{CrI}\;=\frac{I_{002}\;-\;I_{\text{AM}}}{I_{002}}\;\times \;100, \end{array}$$

where *I*_002_ represents the maximum intensity of the 002-lattice diffraction at 2*θ* = 22°, and *I*_AM_ is the intensity of the trough at approximately 2*θ* = 18°, attributed to the scattering intensity of amorphous diffraction.

XANES and XRF measurements were performed at the Canadian Light Source (CLS) using the soft X-ray microcharacterisation beamline (SXRMB). The SXRMB is equipped with a Si (111) double-crystal monochromator with a resolving power of 10000. The powder sample was wrapped by a double-sided, conducting carbon tape and transferred to a vacuum chamber for measurements. The sulphur K-edge XANES spectra were recorded in the surface-sensitive total electron yield (TEY) by measuring the sample drain current and in the bulk-sensitive fluorescence yield (FLY) with an SDD detector. Photon energy of 7200 eV (high enough to excite Fe 1s electrons) was applied to record the XRF spectra using the SDD detector. The energy scale was calibrated by setting the argon K-edge in the upstream ion chamber and a sulphate peak at 2481.6 eV. The data were calibrated and normalised using Athena processing software, and the accuracy of the energy reported here was 0.1 eV.

The pH value was measured using a Mettler Toledo pH meter in accordance with GB/T 6043-2009 [15]. Each sample was immersed in a beaker of deionised water at room temperature. The pH value of the water containing the sample was measured twice to take the average.

The studied samples were analysed on a Bruker FT-IR spectrometer (Alpha) equipped with a diamond crystal (Bruker Optics-Alpha-P) single-reflection attenuated total reflectance (ATR) tool. The spectra were recorded in the spectral range of 4000–400 cm^−1^. The scanning proceeded for 40 successive scans per sample with a resolution of 4 cm^−1^. The data were acquired and normalised using the software OPUS 6.5.

## 3. Results and Discussion

### 3.1. Evaluation of Salt Crystallisation

#### 3.1.1. Micromorphological Analysis

**Figure 2:**
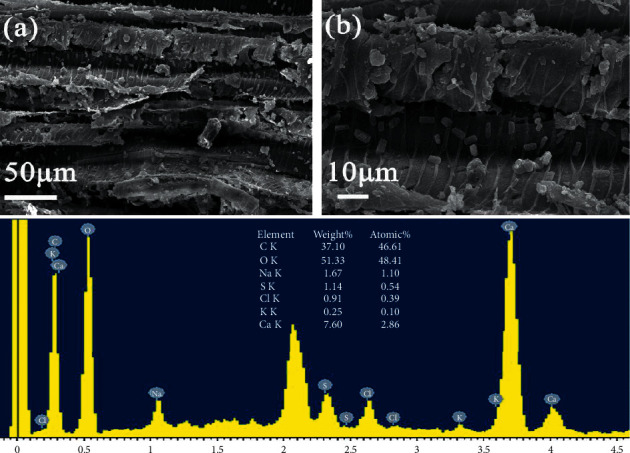
SEM and EDS microanalyses of sample 4F27. (a) Several nonwooden materials attached to the cell wall. (b) Higher-magnification image showing dense, granular solids. (c) Presence of inorganic elements such as sulphur, calcium, sodium, and chlorine.

As shown in the SEM image, several nonwooden materials can be found in the aged wood. These materials are dense, granular solids adhered to the cell wall (Figures 2(a) and 2(b)).  Figure 2(c) shows the elemental composition of the filled solids in sample 4F27, obtained through EDS. Interestingly, in addition to the main constituent elements of wood, namely, carbon and oxygen, the presence of inorganic elements, such as sulphur, calcium, sodium, and chlorine, is detected, as shown in Figure 2(c). Taking into account the trace amounts of inorganic elements, the source of these inorganic elements is foreign; they are not from the original wood. Considering that salt crystals can be composed of these inorganic elements, this indicates the existential possibility of salt in aged wood.

#### 3.1.2. Characterisation of Crystallinity

**Figure 3:**
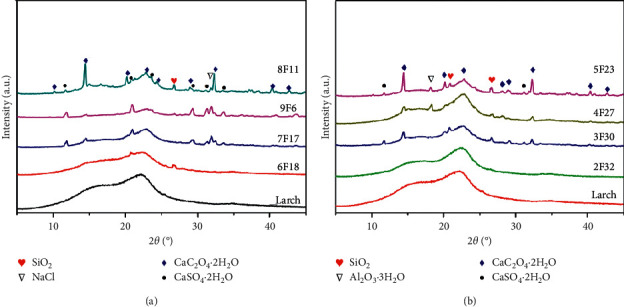
(a) XRD patterns of the aged samples 9F6, 8F11, 7F17, and 6F18 and of the sound sample. Identified phases: SiO_2_, NaCl, CaSO_4_·2H_2_O, and CaC_2_O_4_·2H_2_O. (b) XRD patterns of the aged samples 5F23, 4F27, 3F30, and 2F32 and of the sound sample. Identified phases: SiO_2_, Al_2_O_3_·3H_2_O, CaSO_4_·2H_2_O, and CaC_2_O_4_·2H_2_O.

The crystallinity of the wooden samples was characterised by XRD.  Figure 3 presents the XRD patterns of the aged wood and corresponding sound samples. The major diffraction peaks in the samples of 9F6, 8F11, 7F17, and 6F18 observed at 2*θ* values of 11.6, 20.7, 29.1, 31.2, 32.7, 33.3, 36.1, 40.6, and 43.6° can be indexed to the phase of gypsum, CaSO_4_·2H_2_O (Figure 3(a)). Additionally, peaks at 14.4, 20.2, 22.8, 28.3, 29, 32.4, 40.3, and 42.8° can be identified in several samples (such as 5F23, 4F27, and 3F30, in Figure 3(b)), which can be assigned to the crystal structures of weddellite. Some crystals, such as quartz, gibbsite, and sodium chloride, were also identified on a few aged wood samples. No crystalline hydrated salt can be inferred from the XRD patterns of sample 2F32 nor from those of the sound sample, suggesting that the above crystalline hydrated salt is foreign, rather than from the original wood. The XRD results correspond to the inorganic elements obtained by EDS, further confirming that the above-nonidentified materials are crystalline hydrated salt. Large amounts of crystalline salts, such as CaSO_4_, NaNO_3_, NaCl, and Ca(NO_3_)_2_, have been detected on the surfaces of mural and tracery made of lime-sand mortar in the St. James Church, Liège, and the Postumius Tomb, Roman Necropolis of Carmona, Spain [16, 17].

#### 3.1.3. Type and Amount of Salt

**Figure 4:**
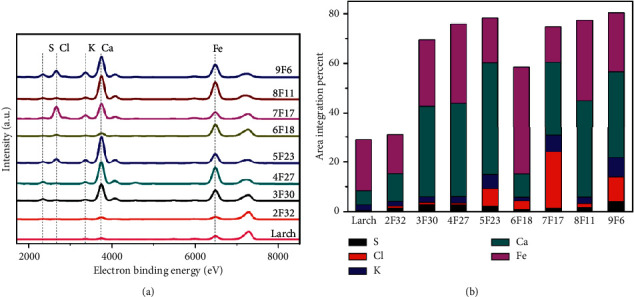
(a) XRF spectra of the aged and sound wood samples, where the well-resolved peaks of sulphur, chlorine, potassium, calcium, and iron ion can be detected. (b) Area integration percent of the elements in the aged and sound wood samples. The aged samples (9F6, 8F11, 7F17, 5F23, and 4F27) contain more inorganic element content than the sound sample, particularly the contents of calcium, sulphur, chlorine, and iron ions.

XRF and XANES analyses were performed on the basis of the above analyses, following the procedures given in [18], to better elucidate the changes in the composition and content of the inorganic elements, chemical environment, and elemental valency of the samples.  Figure 4(a) shows the XRF survey spectra of the investigated wooden samples, giving qualitative information about the inorganic elements. The intensity of each XRF spectrum is normalised to the scatter X-ray peak at 7200 eV, so that it is possible to compare the relative intensity of the different elements. The XRF spectra of the investigated samples were deconvoluted to obtain the height and integral area of the peaks.  Figure 4(b) shows the stacking histogram of the relative percentage graph based on the peak areas of the inorganic elements. As shown in Figures 4(a) and 4(b), potassium, calcium, and iron signals can be detected in the XRF spectrum of the sound sample; wood is reported to contain trace amounts of inorganic elements, typically accounting for 0.3–1.0% of dry wood [19]. Both 6F18 and 2F32 samples were similar to the sound wood sample, with the 6F18 sample having a relatively higher iron intensity.

In the other aged wood samples, the calcium and iron intensities were significantly higher than those in the sound wood sample, and more importantly, additional peaks at lower energy, corresponding to sulphur and chlorine, were detected in the XRF spectra of aged wood samples 9F6, 8F11, 7F17, 5F23, 4F27, and 3F30. These results are consistent with the XRD observation of salt crystallisation, indicating the coexistence of the crystals and ions of salt. Acid rainwater in China contains potassium, sodium, calcium, and chloride ions [20]. Similar inorganic ions were found in the aged wood samples and in the samples (Figures 4(a) and 4(b)). Historic wooden architectures are exposed to complex environmental factors such as UV radiation, water, and atmosphere [21]. Here, atmospheric pollutants and rain, especially acid rain, allow some inorganic substances to diffuse into the wood and form residues [22]. Moreover, chloride can originate from the excrement and decomposition of pigeons [16].

**Figure 5:**
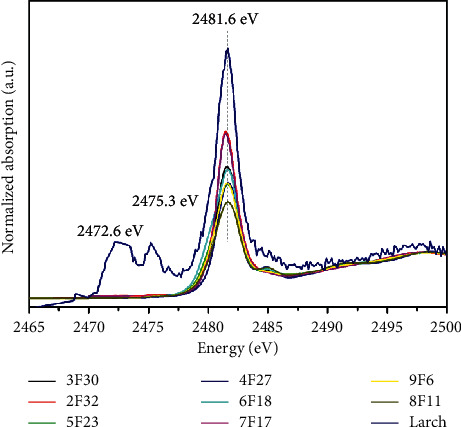
XANES spectra of the aged and sound samples. The dominant peak is sulphate (2481.6 eV), and the two additional peaks at 2472.6 and 2475.3 eV in the sound sample spectrum can be attributed to the reduced forms of sulphur such as aliphatic sulphide and thiophene.

Figure 4(b) shows that the sulphur concentration in the aged wood samples is higher than that in the sound wood sample, as confirmed from the XRF analyses; some sulphate salt can also be detected in the aged wood sample, as confirmed from XRD. However, some organically bound sulphur in the lignin-rich parts of the wood microstructure has been detected through SEM and EDS [23]. Sulphur K-edge XANES spectroscopy has been used to distinguish the chemical forms of sulphur in waterlogged archaeological wood [24]. From Figure 5, several peaks can be observed in the sulphur K-edge XANES spectra of the sound and aged wood samples. The sulphur K-edge spectra of all the wood samples are dominated by a sulphate peak at 2481.6 eV, while there are two additional absorption peaks in the spectrum of the sound wood sample at 2472.6 and 2475.3 eV, which could be assigned to the reduced forms of sulphur such as aliphatic sulphide and thiophene. Moreover, the spectrum of the sound wood sample is noisy, indicating a much lower sulphur content. On the contrary, the absence of the sulphide peak in the aged wood samples suggests the occurrence of oxidation reactions over time. These results are consistent with the increased amount of sulphur in wrecked warships; however, the source of sulphur is different. In shipwrecks, sulphur probably originates from the large quantities of sulphur in reduced forms produced by bacteria in seawater and then transformed to different sulphate salts by oxidation [25]. The surface-sensitive TEY (5 nm) and bulk-sensitive FLY (50 nm) spectra of the samples have the same spectral characteristics, demonstrating the stability of these samples under ambient conditions.

A significant amount of sulphate was detected in the aged wood samples and small amounts of sulphide in the sound wood sample, as confirmed by the sulphur K-edge XANES spectrum; these can be assumed to be due to the oxidation of sulphide from the original sound wood and/or the accumulation of sulphate of invasive nature. A stepwise oxidisation reaction of sulphide from the original wood under oxygen-rich and humid conditions could clearly produce sulphate:

(2) $$\begin{array}{c} {\text{S}}^{2-}{+\text{O}}_{2}{+2\text{H}}_{2}\text{O}⟶{4\text{H}}^{+}\left(\text{aq}\right){+\text{SO}}_{4}^{2-} \end{array}$$

Considering the higher content of sulphur in the aged wood samples, the source of sulphate in aged wood may still be invasive accumulation mainly affected by environmental factors such as rain and atmosphere. Yingxian Wooden Pagoda is located near Datong, which has one of the largest coal resources in China. With the massive development of the coal mining industry in Datong and the utilisation of coal in the region, large amounts of sulphur dioxide and nitrogen oxides are emitted, leading to poor air quality in the north of Shanxi province area [26]. Gaseous pollutants can react with porous building materials, leading to the formation of sulphate salt [16].

### 3.2. Salt-Induced Damage

#### 3.2.1. Mechanical Damage to Aged Wood

**Figure 6:**
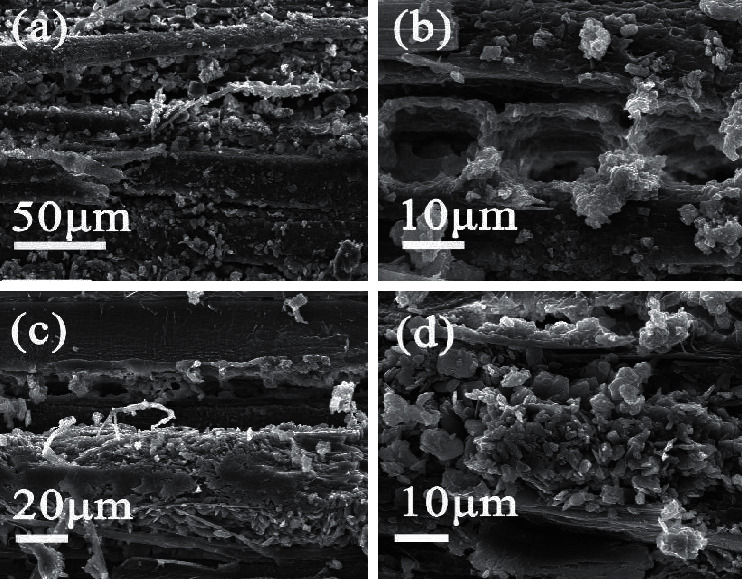
(a–d) SEM images of sample 9F6, where massive salt crystals fill the cell walls.

The salt-induced damage of building materials is a recognised form of deterioration, and its common mechanism is mechanical damage to the materials [27]. The SEM observation revealed that all the Yingxian Wooden Pagoda samples have been degraded to different degrees. As shown in Figure 6, in the sample 9F6, massive salt crystals fill almost the entire rays and lumen of the cell walls, causing tracheid and ray cell deformation and bursting the wood cell walls. Salt crystallisation is through the crystallisation-dissolution cycle. When the inorganic ions were transformed into inorganic salts with low solubility, as exemplified in reactions (3) and (4), salt is formed on the surface of the wood. Hime et al. showed that when an anhydrous salt imbibes moisture and undergoes solid-state hydration, its volume expands, leading to rupture and severe mechanical damage to the material structure [28]. Moreover, due to the drastic cyclic change between day and night temperatures, salt hydration and crystallisation produce pressures much higher than the strength of the material, resulting in material damage [5].

(3) $$\begin{array}{c} {\text{Ca}}^{2+}{+\text{nH}}_{2}{\text{O}+\text{SO}}_{4}^{2-}⟶{\text{CaSO}}_{4}\cdot {\text{nH}}_{2}\text{O}\left(\text{s}\right) \end{array}$$

(4) $$\begin{array}{c} {\text{C}}_{2}{\text{O}}_{4}^{2-}{+\text{Ca}}^{2+}{+\text{nH}}_{2}\text{O}⟶{\text{CaC}}_{2}{\text{O}}_{4}\cdot {\text{nH}}_{2}\text{O}\left(\text{s}\right) \end{array}$$

#### 3.2.2. Chemical Damage to Aged Wood

**Figure 7:**
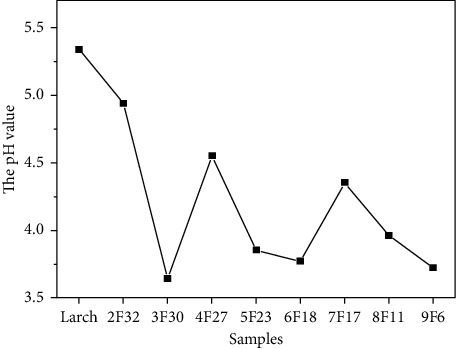
Results of pH measurement of the aged and sound wood samples. The acidity of each aged wood sample is higher than that of the sound sample.

Reported studies show that high-concentration inorganic elements, such as iron, silicon, and sulphur, penetrate warship wrecks, leading to a decrease in pH [29]. Sulphate radical is the main acidogenic substance in the rainwater in China [20].  Figure 7 shows the pH measurements of the investigated wooden samples. The pH values in the aged wood samples ranged from 4.94 to 3.64, which is significantly lower than that of the sound sample (5.34). The apparent increase in the acidity of the aged wood samples is consistent with that observed by Capano et al. who studied wooden artefacts discovered in the Samnite Sanctuary [30]. In this case, the higher acidity in the aged wood samples can be attributed to inorganic substances.

**Figure 8:**
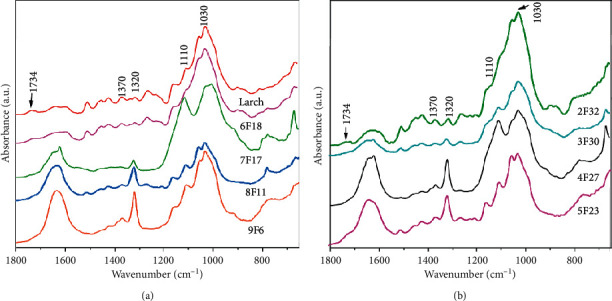
(a) FT-IR spectra of the sound wood sample (larch) and of aged wood samples 9F6, 8F11, 7F17, and 6F18. (b) FT-IR spectra of the samples 5F23, 4F27, 3F 30, and 2F 32. The peaks of polysaccharides are highlighted by indicating the wavenumber.

Because of the slightly high acidity of the aged wood samples, the changes in their chemical compositions need to be further determined. In most cases, lignin is considered the most resistant wood component against acid agents while cellulose and hemicelluloses are more susceptible to degradation [21]. ATR FT-IR spectroscopy can be employed to qualitatively identify the structure of wood components and their molecular functional groups. The ATR FT-IR spectra of most aged wood samples present characteristic peaks of cellulose, while the bands of hemicelluloses disappear, as shown in Figure 8. The bands at 1026, 1110, 1320, 1370, and 1734 cm^−1^ can be attributed to polysaccharides [31]. Among them, the band at 1734 cm^−1^ (ester group, with C=O bond) is a characteristic peak of hemicellulose and is well-evident in the sound wood sample (Figure 8(a)). The peak almost disappears in the aged wood samples, confirming the degradation of hemicelluloses. These alternations can be explained by the lower stability of hemicellulose because its structure consists of several different types of low molecular weight, highly branched monosaccharides, and less ordered crystal structure [32].

Although the spectrum qualitatively proves the presence of cellulose in the aged wood samples, its degree of degradation remains unknown. The crystallinity (CrI) of cellulose was further analysed by XRD, i.e., the relative proportion of crystallinity regions in cellulose. This is because a cellulose chain contains crystalline, semicrystalline, and amorphous regions.  Table 1 lists the CrI values of the investigated samples, where ∆CrI represents the variation in the properties of the aged wood sample compared to the sound sample. The CrI value of the sound sample is 28%, which falls in the CrI value range (24–31%) for nondecayed wood [33]. A significant CrI increase tendency is observed in most of the aged samples compared to the sound sample, with samples 9F6 and 7F17 showing more than double the value. This can be attributed to the degradation of aged wood, which decreases the content of the amorphous regions in the cellulose, consequently increasing the overall CrI content. In fact, amorphous regions exhibit lower density than crystalline regions in the fibre, making them more susceptible to chemical reactivity and preferential degradation [34]. A similar trend has been found for wood samples, where the CrI value increased significantly during the decay process [35]. When the amorphous regions of the cellulose crystals completely degrade, the cellulose crystal regions begin to degrade. Such an initial increase is generally followed by a further rapid decrease in CrI [36].

The accumulation of sulphate in the aged wood might be the cause of carbohydrate degradation. Acidogenic substances are products of oxidation reaction and rainwater, accompanied by the production of plenty of hydrogen ions [23]. Thus, acidogenic substances may be produced in the aged wood samples via the following chemical reaction:

(5) $$\begin{array}{c} {2\text{SO}}_{2}{+\text{O}}_{2}{+2\text{H}}_{2}\text{O}⟶{4\text{H}}^{+}\left(\text{aq}\right){+2\text{SO}}_{4}^{2-} \end{array}$$

The presence of hydrogen ions, which accelerate degradation, shown in reactions (6) and (7), would eventually cause the cleavage of the *β*-glycosidic bond linking the D-glucopyranose units and decrease the degree of polymerisation of cellulose and hemicelluloses [37]. The quantity and quality of cellulose fibres have an important effect on the properties and stability of the wood. These would lead to the crushing and distortion of aged wood samples, thus significantly reducing the mechanical stability of the wood structure. Simultaneously, a higher degree of iron ion and sodium chloride crystallisation was found in the aged wood samples as observed by XRF and XRD. Under aerobic and humid conditions, iron can act as a catalyst, accelerating the oxidation of sulphur in the reduced state (sulphide) to oxidised states (sulphates and sulphuric acids) and leading to the degradation of organic materials in the wood [38]. The presence of sodium chloride can improve the solubility of gypsum, thereby increasing the salt mobility and crystallisation on the surface [11].

(6) $$\begin{array}{c} \left({\text{C}}_{6}{\text{H}}_{10}{\text{O}}_{5}\right){\text{n}+\text{nH}}_{2}\text{O}\underset{⟶}{{\text{H}}^{+}}{\text{nC}}_{6}{\text{H}}_{12}{\text{O}}_{6} \end{array}$$

(7) $$\begin{array}{c} \left({\text{C}}_{6}{\text{H}}_{8}{\text{O}}_{4}\right){\text{n}+\text{nH}}_{2}\text{O}\underset{⟶}{{\text{H}}^{+}}{\text{nC}}_{5}{\text{H}}_{10}{\text{O}}_{5} \end{array}$$

**Figure 9:**
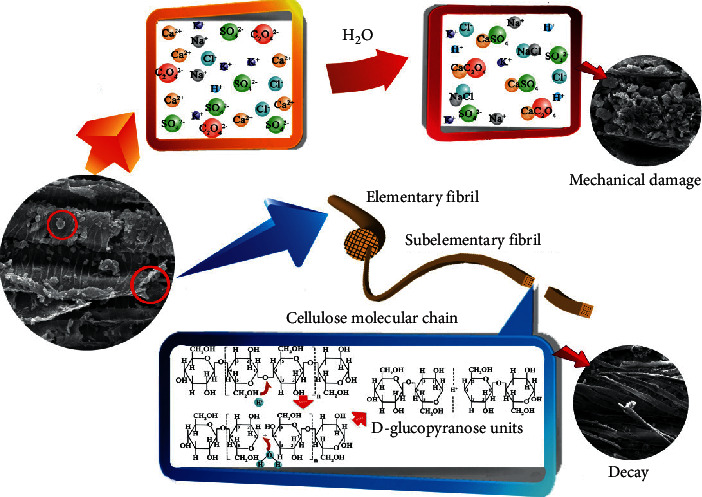
Unusual corrosion mechanism in aged wood. The arrows indicate two corrosion routes: the hydrolysis of carbohydrates due to acidogenic substances from the atmosphere and the mechanical damage of the cell structure due to salt formation.

Therefore, because of the above effects induced by salt on the aged wood (Figure 9), relevant treatments should be developed in future studies. First, the pH values and humidity at different positions in Yingxian Wooden Pagoda should be monitored over a long period. Second, some chelating agents can be applied to form soluble substances with iron and thereby remove iron from the wood. Finally, exploring agents that are harmless to wood is suggested; these could form a protective layer on the surface of aged wood to prevent deterioration from external oxygen, rain, and other factors.

## 4. Conclusion

In this study, multiple chemical characterisation methods were applied to evaluate the salt-induced damage of Yingxian Wooden Pagoda, a typical wooden historical architecture, including the nature and damage patterns. The following are the conclusions drawn from the study:

1. Through micromorphological observation, massive dense, granular materials were found in the aged wood samples. The EDS spectrum indicated that these materials contain inorganic elements, such as sulphur, calcium, sodium, and chlorine, consistent with the detection of some crystalline hydrated salts, such as gypsum and wollastonite, by XRD. Therefore, the presence of salts was confirmed in the aged wood of Yingxian Wooden Pagoda
2. An increase in the content of ions, such as sulphur and calcium ions, was identified by XRF. XANES spectroscopy analysis confirmed that the element sulphur in the aging wood was in the form of sulphate, indicating that environmental factors, such as acid rain and atmospheric pollutant, are the main sources of invasive inorganic elements, leading to the accumulation of inorganic salts
3. Two damage mechanisms were proposed. A pH characterisation of the aged wood samples showed that these samples exhibit a higher acidity than the sound wood sample. The ATR FT-IR spectrum of the aged wood sample showed that the hemicellulose peak almost disappears, evidencing its degradation, and showed a significant increase in CrI, further indicating a preferential degradation of the amorphous regions in cellulose. Therefore, it can be concluded that the presence of acidogenic substances from the atmosphere, such as the accumulation of sulphate salts, would eventually cause the hydrolysis of carbohydrates. On the other hand, massive salt crystallisations and ions were observed inside the aged wood, resulting in mechanical damage to the wood structure. Finally, some treatments and corresponding protective measures were recommended based on the damages.

This interdisciplinary research, combined with systematic and quantitative multichemical analysis methods, provides new insights into the effect of salt damage in historic wooden buildings. The results can aid in decision making to minimise further damage and may also help in evaluating other similar historical buildings.

## Figures and Tables

**Table 1:** CrI and ∆CrI of the sound and aged wood samples.

Samples	Larch	9F6	8F11	7F17	6F18	5F23	4F27	3F30	2F32
CrI (%)	28	56	49	63	26	52	50	49	30
∆CrI (%)	—	100	75	125	−7	86	79	75	7

## Data Availability

The data used to support the findings of this study are included within the article.

## Supplementary Materials

Supplementary Materials Figure S1: (a) appearance of Yingxian Wooden Pagoda. The total height is 67.31 m and the bottom diameter is 30.3 m. (b) The inner structure of Yingxian Wooden Pagoda. A multistoreyed wooden framework pagoda has five outer storeys and four mezzanine storeys. Figure S2: (a) sampling position of aged wood on the 3^rd^, 5^th^, 7^th^, and 9^th^ floors. (b) Sampling position of aged wood on the 2^nd^, 4^th^, 6^th^, and 8^th^ floors.
