# Supplementary material for: Evaluation of Salt-Induced Damage to Aged Wood of Historical Wooden Buildings
Source: Int J Anal Chem. 2020 Aug 1;2020:8873713. doi: 10.1155/2020/8873713 (PMC7416263; doi:10.1155/2020/8873713)
Supplement: Supplementary Materials — Figure S1: (a) appearance of Yingxian Wooden Pagoda. The total height is 67.31 m and the bottom diameter is 30.3 m. (b) The inner structure of Yingxian Wooden Pagoda. A multistoreyed wooden framework pagoda has five outer storeys and four mezzanine storeys. Figure S2: (a) sampling position of aged wood on the 3rd, 5th, 7th, and 9th floors. (b) Sampling position of aged wood on the 2nd, 4th, 6th, and 8th floors. [file 8873713.f1.docx]

**Evaluation of salt-induced damage to aged wood of historical wooden buildings**

Xiaochen Mi ^a^, Tieying Li ^a^*, Jinping Wang ^b^, Yongfeng Hu *^c^*

*^a^ Collage of Civil Engineering, Taiyuan University of Technology, Taiyuan 030024, Shanxi, P. R. China*

*^b^ Collage of Architecture, Taiyuan University of Technology, Taiyuan 030024, Shanxi, P. R. China*

*^c^ Canadian Light Source, 44 Innovation Boulevard, Saskatoon, SK S7N 2V3, Canada*

* **Corresponding author:**

*E-mail address:* [tieyingli11@163.com](mailto:tieyingli11@163.com)

**Supplementary material:**

**1. Introduction**


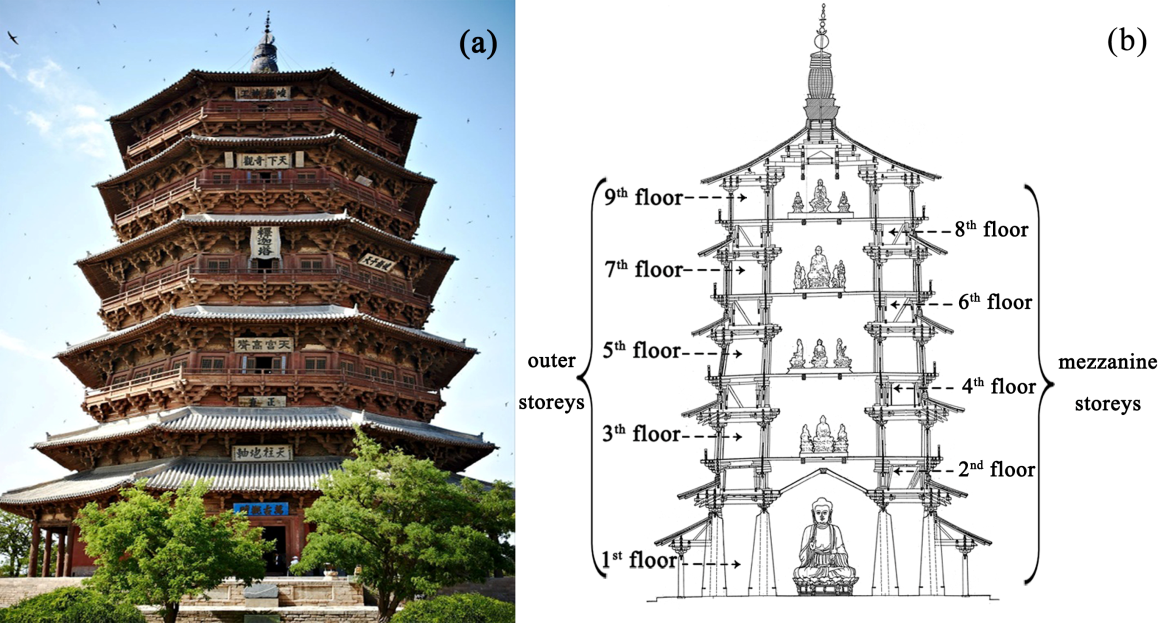


Fig.S1 (a) Appearance of Yingxian Wooden Pagoda. A total height is 67.31m and a bottom diameter is 30.3 m. (b) Inner structure of Yingxian Wooden Pagoda. A multi-storeyed wooden framework pagoda has five outer storeys and four mezzanine storeys.

Yingxian wooden pagoda is a multi-storeyed wooden framework pagoda, with a total height of 67.31m and a bottom diameter of 30.3 m. It consists of nine layers with six eaves but, interestingly, from the outside it seems that the pagoda only has five storeys(Fig. S1a). This can be explained by the fact that Yinxian wooden pagoda has five outer storeys and four mezzanine storeys, namely, there is a hidden layer between each outer story (Fig. S1b).

**2. Samples**

**
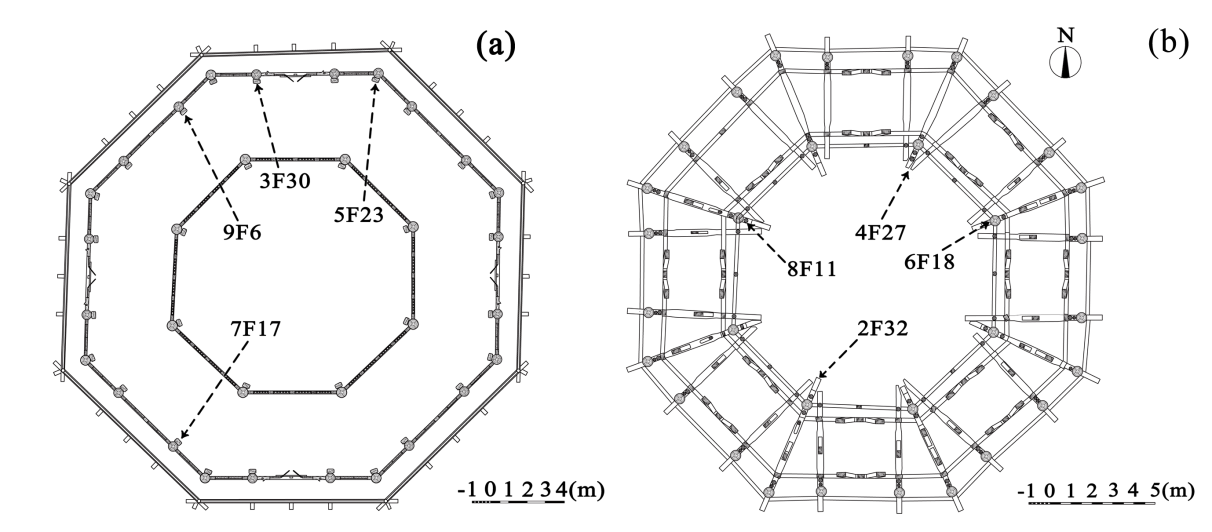
**

Fig. S2. (a) Sampling position of aged wood on the 3^rd^, 5^th^, 7^th^, and 9^th^ floors. (b) Sampling position of aged wood on the 2^nd^, 4^th^, 6^th^, and 8^th^ floors.

Sample 9F6, 7F17, 5F23, 3F30 and 8F11, 6F18, 4F27, 2F32 were taken from the outer storeys and the mezzanine storeys, respectively, which is the surface of upright pillars from different positions in the second to the ninth layers of Yingxian Wooden Pagoda.
